# Cancer risks in people on dialysis and kidney transplant recipients: a Catalan cohort study, 2003–21

**DOI:** 10.1093/ckj/sfaf077

**Published:** 2025-03-20

**Authors:** Laia Oliveras, Laura Pareja, Josepa Ribes, Jordi Comas, Carlos Couceiro, Àlex Favà, Sergi Codina, Ana Coloma, Anna Manonelles, Nuria Lloberas, Edoardo Melilli, Elisenda Martinez-Carbonell, Jordi Gálvez, Sonia Mosteiro, Jaume Tort, Josep M Borràs, Josep M Cruzado, Nuria Montero

**Affiliations:** Hospital Universitari de Bellvitge, Nephrology Department, L'Hospitalet de Llobregat, Spain; Biomedical Research Institute (IDIBELL), Hospital Duran i Reynals, Barcelona, Spain; Facultat de Medicina i Ciències de la Salut, Universitat de Barcelona (UB), Barcelona, Spain; Hospital Cancer Registry Unit, Catalan Institute of Oncology, Barcelona, Spain; Department of Public Health, Mental Health and Maternal and Child Health Nursing, Faculty of Nursing, University of Barcelona, Barcelona, Spain; Hospital Cancer Registry Unit, Catalan Institute of Oncology, Barcelona, Spain; Catalan Pathology Registry, Catalan Cancer Plan, Department of Health of Catalonia, Barcelona, Spain; Department of Clinical Sciences, University of Barcelona (UB), Barcelona, Spain; Catalan Transplant Organization, Department of Health of Catalonia, Barcelona, Spain; Hospital Universitari de Bellvitge, Nephrology Department, L'Hospitalet de Llobregat, Spain; Biomedical Research Institute (IDIBELL), Hospital Duran i Reynals, Barcelona, Spain; Hospital Universitari de Bellvitge, Nephrology Department, L'Hospitalet de Llobregat, Spain; Hospital Universitari de Bellvitge, Nephrology Department, L'Hospitalet de Llobregat, Spain; Biomedical Research Institute (IDIBELL), Hospital Duran i Reynals, Barcelona, Spain; Hospital Universitari de Bellvitge, Nephrology Department, L'Hospitalet de Llobregat, Spain; Hospital Universitari de Bellvitge, Nephrology Department, L'Hospitalet de Llobregat, Spain; Biomedical Research Institute (IDIBELL), Hospital Duran i Reynals, Barcelona, Spain; Biomedical Research Institute (IDIBELL), Hospital Duran i Reynals, Barcelona, Spain; Hospital Universitari de Bellvitge, Nephrology Department, L'Hospitalet de Llobregat, Spain; Biomedical Research Institute (IDIBELL), Hospital Duran i Reynals, Barcelona, Spain; Agency for Health Quality and Assessment of Catalonia, Department of Health of Catalonia, Barcelona, Spain; Hospital Cancer Registry Unit, Catalan Institute of Oncology, Barcelona, Spain; Hospital Cancer Registry Unit, Catalan Institute of Oncology, Barcelona, Spain; Catalan Transplant Organization, Department of Health of Catalonia, Barcelona, Spain; Catalan Pathology Registry, Catalan Cancer Plan, Department of Health of Catalonia, Barcelona, Spain; Department of Clinical Sciences, University of Barcelona (UB), Barcelona, Spain; Hospital Universitari de Bellvitge, Nephrology Department, L'Hospitalet de Llobregat, Spain; Biomedical Research Institute (IDIBELL), Hospital Duran i Reynals, Barcelona, Spain; Hospital Universitari de Bellvitge, Nephrology Department, L'Hospitalet de Llobregat, Spain; Biomedical Research Institute (IDIBELL), Hospital Duran i Reynals, Barcelona, Spain

**Keywords:** cancer epidemiology, cancer risks, dialysis, kidney failure, kidney transplant

## Abstract

**Background:**

People with kidney failure have a higher risk of cancer compared with age- and sex-matched individuals in the general population, yet data from southern Europe are limited. This study explores cancer incidence in the kidney failure population in Catalonia.

**Methods:**

We identified cancer cases through linkage of the Catalan Kidney Registry with Catalan cancer databases. Standardized incidence ratios (SIRs) were calculated for all-site and site-specific cancers in people on dialysis and kidney transplant recipients.

**Results:**

We described the epidemiology of cancer in 21 595 people on dialysis and 8037 kidney transplant recipients in Catalonia (2003–21). Cancer risk was more than two times higher in people on dialysis (SIR 2.11, 95% CI 2.02–2.19) and nearly four times higher in kidney transplant recipients (SIR 3.82, 95% CI 3.65–3.99) compared with the general population. Risks varied by cancer site, with a significantly higher incidence of kidney and thyroid cancers in the dialysis cohort, and skin cancer in the transplant cohort. The highest cancer risks were observed in the youngest, those with glomerular diseases, and those with the longest time since transplantation.

**Conclusions:**

People with kidney failure face a high burden of cancer, particularly after kidney transplantation. Understanding the epidemiology of cancer in the kidney failure population is crucial for shaping health policies.

KEY LEARNING POINTS **What was known:**

- People on dialysis and kidney transplant recipients have a higher risk of cancer compared with the general population.
- There is little research focusing on cancer risks in the kidney failure population of southern Europe, with limited cancer cases and lack of accounting for non-melanoma skin cancer.
- Differences in healthcare systems, treatment practices, or environmental exposures can impact the cancer risk patterns in our region.

**This study adds:**

- Cancer risk was more than 2-fold higher for people on dialysis and nearly 4-fold higher after kidney transplantation compared with the general population. Risks varied by cancer site.
- The highest excess cancer risk was observed in the youngest individuals, those with glomerular diseases, and those with the longest time since transplantation.
- Compared with other regions, melanoma showed more than double the excess risk in our kidney failure population.

**Potential impact:**

- We present a large and contemporary cohort describing cancer risks in the dialysis and kidney transplant population in southern Europe.
- Understanding the epidemiology of cancer in the kidney failure population of our region is crucial for identifying individuals at higher risk of cancer and shaping tailored health policies.

## INTRODUCTION

The burden of chronic kidney disease is increasing, with >70 000 people starting kidney replacement therapy in Europe in 2021 [1]. Despite improvements in dialysis techniques, organ procurement procedures and immunosuppression regimens, people with kidney failure still face significant challenges from comorbidities, such as cardiovascular disease and cancer [1].

People on dialysis have ∼1.5 times the risk of developing cancer compared with age- and sex-matched individuals in the general population. This risk increases 2- to 4-fold after kidney transplantation. During dialysis, the most significant increases in cancer incidence compared with the general population are seen in kidney, urinary tract, and thyroid cancers [2, 3]. After kidney transplantation, the highest risks are associated with Kaposi sarcoma, non-melanoma skin cancers, post-transplant lymphoma, and anogenital cancers [2, 3].

There is considerable variability among studies regarding cancer risks in the population with kidney failure [4–7]. This variability may be attributed to methodological differences, such as how cancer diagnoses are identified, as well as differences in the management of chronic kidney disease, access to medical care, or environmental exposures.

Few studies have focused on the epidemiology of cancer for the kidney failure population in southern Europe, often reporting a small number of cancer events and not accounting for non-melanoma skin cancer [8, 9]. While other large national, population-based cohorts have already reported cancer risks in the kidney failure population, these findings may not be entirely generalizable to southern Europe.

Catalonia is a region in northeastern Spain, with a public healthcare system and a high kidney donation rate of 90–120 donors per million of population in the past 5 years [10]. In 2022, 949 kidney transplants were performed, and around 1300 people started some type of dialysis treatment [10]. The most common cancers in the general Catalan population are prostate, colorectal, and lung cancers [11]. Understanding the specific risks in the kidney failure population can have potential policy implications.

The aims of this study were (i) to describe the incidence of cancer in people on dialysis and kidney transplant recipients compared with the general population in Catalonia, and (ii) to identify those with the highest excess risk of cancer.

## MATERIALS AND METHODS

### Study population and design

We conducted a population-based study in Catalonia to describe the incidence of cancer in the kidney failure population compared with the expected incidence in the general population.

The population with kidney failure was obtained from the Catalan Kidney Registry. This is a population-based registry with mandatory notification that collects data since 1984 from all dialysis and transplant centers in Catalonia. It includes information on kidney replacement therapies, comorbidities, and patient outcomes. External validation showed exhaustive reporting on variables and excellent concordance [12]. We included all adults and children who started kidney replacement therapy between 2003 and 2021, regardless of their previous history of cancer.

Follow-up for the dialysis population started on the date of the first dialysis until the earliest occurrence of invasive cancer, death, kidney transplant, or 31 December 2021. For kidney transplant recipients, follow-up began on the date of the first transplant and continued until the earliest occurrence of cancer diagnosis, death, or 31 December 2021, without censoring for graft failure or successive transplants. People who received a kidney transplant after being on dialysis were censored from the dialysis cohort, and their follow-up time started to contribute to the transplant cohort.

Patients were not excluded based on their history of cancer before starting kidney replacement therapy. However, any cancers that occurred within the first 2 months after the start of dialysis or kidney transplantation were excluded from the analysis to minimize the misclassification of previous cancers as new cancers. After cancer diagnosis, patients did not contribute follow-up time for that specific cancer type; however, they continued to contribute follow-up time for other cancer types.

### Cancer diagnoses

The Catalan Kidney Registry collects data on cancer diagnoses, including date and topography, based on notifications from dialysis and transplant centers. To ensure the completeness of the cancer data, we linked it to four additional cancer databases using a unique personal identification number:

(i) The Registry of Hospital Tumors of the Catalan Institute of Oncology is a multicenter hospital-based cancer registry that collects data from six large tertiary hospitals that cover a population of >2.5 million inhabitants. It is the largest hospital cancer registry in Catalonia. Data on cancer diagnosis is thorough, with the participation of expert documentation technicians in cancer registries. Cancer diagnoses are coded with the International Classification of Diseases for Oncology, 3rd edition, 2nd revision (ICD-O-3.2) [13]. Data have been collected since 2013, and we linked data for the period 2013–21.
(ii) The Pathology Registry is a real-time population-based registry of the Catalan Department of Health that captures diagnoses from pathology laboratories in public hospitals in Catalonia, coded in SNOMED-CT terminology [14]. Data have been collected since 2017, and record linkage was performed for the period 2017–21.
(iii) The Minimum Basic Data Set is a population-based registry of the Catalan Department of Health that collects information on discharge records from all hospitals in Catalonia since 2005, coded using the International Classification of Diseases (ICD-9th or ICD-10th version depending on calendar year) [15, 16]. This data source provided data from 2006 to 2021.
(iv) The Primary Care Clinic Station is an electronic health record system that collects data used by healthcare and social care professionals in primary care centers and outpatient facilities, coded using the International Classification of Diseases (ICD-10th version) [16]. This data source provided data for the period 2003–21.

Data linkage was performed by the Catalan Kidney Registry of the Catalan Department of Health, and we obtained data through de-identified information.

Data validation was conducted with the Registry of Hospital Tumors team in accordance with international regulations for population-based cancer registries [17]. The topography and morphology of the invasive cancer diagnoses were mapped to the ICD-O-3 [13]. Only invasive cancers (C00–C80) were included in the study.

If tumor sites matched across different data sources, the earliest date was collected as the incidence date. In case of different tumor sites, data from the Registry of Hospital Tumors were prioritized for decision making due to its thoroughness. If no data from the Registry of Hospital Tumors were available, they were considered multiple tumors, and the earliest incidence date was collected for each tumor.

### Statistical analysis

Demographic and clinical characteristics were summarized using absolute counts and proportions, and follow-up using person-years.

For the study period, age- and sex-specific cancer incidence rates for the general population in Catalonia were obtained from the Catalan Cancer Plan of the Department of Health [11]. These rates are calculated using data from the Girona and Tarragona population-based cancer registries. A Bayesian modeling approach is applied using temporal autoregressive models that account for the effects of age, period of diagnosis, and birth control. These rates are considered representative of the entire Catalan population [11].

We estimated cancer standardized incidence ratios (SIRs) for people on dialysis and kidney transplant recipients, using the general population as the reference group. Confidence intervals for the SIRs were estimated based on the Poisson distribution. The SIR is the ratio of observed cancers in the study population compared with the expected number of cancers in the sex-, age-, and calendar-year-matched general population. A SIR >1 indicates an excess risk compared with the general population [18]. We calculated SIRs for all-site cancer, all-site cancer excluding non-melanoma skin cancer, and site-specific cancers. We also calculated SIRs by dialysis type (hemodialysis and peritoneal dialysis). To identify populations with the highest excess risk, we further estimated SIRs for all-site cancer excluding non-melanoma skin cancer by sex, age, dialysis or transplant era (before or after 2013), comorbidities at the start of kidney replacement therapy, time since dialysis or kidney transplant, and type of immunosuppression received during the first 6 weeks after transplantation.

Data were analyzed using STATA version 17 (Stata Corporation, College Station, USA) and R statistical software. The study was approved by the Research Ethics Committee of the Hospital Universitari de Bellvitge (PR191/22). The study followed the Strengthening the Reporting of Observational Studies in Epidemiology (STROBE) guidelines.

## RESULTS

### Study population and demographics

A total of 21 595 people started dialysis between 2003 and 2021, with 60 307 person-years of follow-up (Table 1). Approximately two-thirds were male, and the median age was 69 years (IQR 57–77). Thirty-seven per cent had diabetes, 18% obesity, and 24% a history of cancer before starting dialysis. During the study period, they developed 2448 cancers (1622 excluding non-melanoma skin cancer). The median time from the start of dialysis to cancer diagnosis was 2 years (IQR 0.99–3.86).

**Table 1:** Demographic and clinical characteristics of people on dialysis and kidney transplant recipients from 2003 through 2021.

	Dialysis cohort			Kidney transplant cohort		
	*n*	%	Person-years	*n*	%	Person-years
Total	21 595	100	60 306.78	8037	100	47 998.64
Sex						
Men	14 175	65.6	38 644.19	5196	64.7	30 208.56
Women	7420	34.4	21 662.59	2841	35.3	17 790.07
Age at start of dialysis or at first transplant (years)						
0–40	1540	7.1	3713.75	1334	16.6	11 036.13
41–60	4781	22.1	12 810.24	3105	38.6	20 491.32
61–75	7947	36.8	22 825.26	3024	37.6	14 739.19
>75	7327	33.9	20 957.53	574	7.1	1731.99
Period						
2003–12	10 342	47.9	36 607.33	2940	36.6	29 095.30
2013–21	11 253	52.1	23 699.44	5097	63.4	18 903.34
Primary kidney disease						
Glomerular	2901	13.4	7694.42	1819	22.6	12 351.81
Diabetes	4887	22.6	14 183.80	1190	14.8	5519.77
Hypertension/renal artery disease	3025	14.0	9270.87	803	10.0	4629.18
Tubulointerstitial/urologic	1664	7.7	5103.05	762	9.5	5007.43
Polycystic kidney disease	1349	6.2	3410.15	1115	13.9	7692.76
Unknown cause/other	6967	32.3	10 445.19	2083	25.9	11 807.61
*Missing*	*802*	*3.7*	10 199.3	*265*	*3.3*	*990.08*
Previous cancer						
Yes	5080	23.5	13 412.17	907	11.3	3705.24
No	16 515	76.5	46 894.61	7130	88.7	44 293.41
Body mass index						
<24.9	8158	37.8	22 127.89	3324	41.4	21 590.74
25–29.9	7363	34.1	21 251.50	2655	33.0	15 521.75
≥30	4174	19.3	12 436.79	1416	17.6	7291.20
*Missing*	*1900*	*8.8*	4490.60	*642*	*7.9881*	3594.95
Diabetes						
Yes	8043	37.2	22 535.87	1769	22.0	7741.21
No	12 529	58.0	35 593.95	5890	73.3	37 893.76
*Missing*	*1023*	*4.8*	2176.96	*378*	*4.7*	2363.67
Time since start of dialysis						
<1 year	4413	20.4	2077.82	N/A	N/A	N/A
1–3 years	5741	26.6	10 438.58	N/A	N/A	N/A
≥3 years	11441	53.0	47 790.38	N/A	N/A	N/A
Time on dialysis prior to transplantation						
<1 year	N/A	N/A	N/A	3055	38.0	20 197.72
1–4 years	N/A	N/A	N/A	3974	49.4	22 612.67
>4 years	N/A	N/A	N/A	1008	12.5	5188.56
Time since first transplant						
<1 year	N/A	N/A	N/A	992	12.3	455.83
≥1 year and <5 years	N/A	N/A	N/A	3051	38.0	9056.33
≥5 years	N/A	N/A	N/A	3994	49.7	38 486.48
Immunosuppression						
Polyclonal antibodies						
Yes	N/A	N/A	N/A	2165	26.9	11 093.78
No	N/A	N/A	N/A	5258	65.4	33 955.62
*Missing*				*614*	*7.6*	
Calcineurin inhibitors						
Yes	N/A	N/A	N/A	6875	85.5	40 475.54
No	N/A	N/A	N/A	561	7.0	4614.82
*Missing*				*601*	*7.5*	
mTORis						
Yes	N/A	N/A	N/A	965	12.0	5193.91
No	N/A	N/A	N/A	6411	79.8	39 303.91
*Missing*				*661*	*8.2*	
No. of cancers	2448			2013		
No. of cancers (excluding NMSC)	1622			975		
Median time to cancer (excluding NMSC), years (IQR)	2.00	(0.99–3.86)		5.00	(2.32–8.87)	

IQR, interquartile range; NMSC, non-melanoma skin cancer.

The kidney transplant cohort comprised 8037 recipients, with 47 998 person-years of follow-up. Compared with the dialysis cohort, they were younger (median age of 57 years, IQR 46–67), and had a lower proportion of people with diabetes (22.0%) and previous cancers (11.3%). During follow-up, they developed 2013 cancers (975 excluding non-melanoma skin cancer). The median time from kidney transplant to cancer diagnosis was longer, at 5 years (IQR 2.32–8.87).

### Standardized incidence ratios for all-site and site-specific cancers

**Figure 1:**
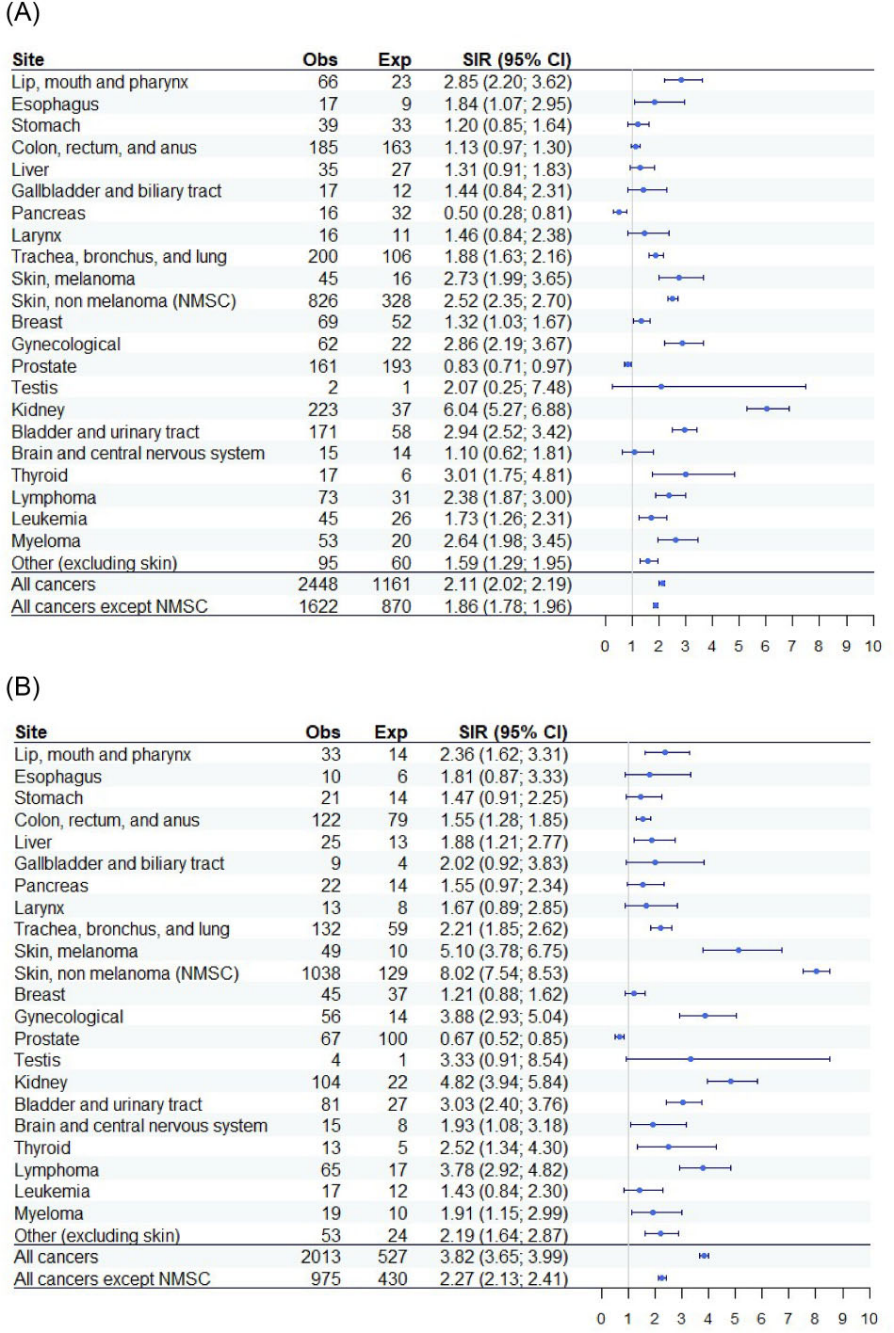
Risk of all-site and site-specific cancer in people on dialysis (**A**) and kidney transplant recipients (**B**) compared with the general population.

In the dialysis cohort, cancer incidence was over two times higher than in the general population (SIR 2.11, 95% CI 2.02–2.19). Excluding non-melanoma skin cancer, the excess cancer risk was reduced, but the incidence of cancer was still significantly higher than in the general population (SIR 1.86, 95% CI 1.78–1.96). Excess risks varied by site: incidence was significantly increased for kidney, bladder and urinary tract, thyroid, melanoma, non-melanoma skin cancer, myeloma, and oral cavity, and pharynx cancer. Conversely, the observed incidence was lower than that expected in the general population in prostate and pancreas cancer. Risks for other sites were similar to the general population (Fig. 1). There were no significant differences between hemodialysis and peritoneal dialysis (Supplementary Table 1).

In the kidney transplantation cohort, cancer risk increased even further, with an overall cancer risk nearly four times higher than that of the general population (SIR 3.82, 95% CI 3.65–3.99). After excluding non-melanoma skin cancer, the risk decreased but still remained more than twice as high as expected (SIR 2.27, 95% CI 2.13–2.41). The highest excess risk was observed for skin cancers, with melanoma occurring more than five times and non-melanoma skin cancer more than eight times as frequently as in the general population. Risks for lymphoma, kidney, bladder and urinary tract, and gynecological cancers were three to five times higher than expected. Only the incidence of prostate cancer was lower than expected, while risks for other sites were more than two times higher than expected.

### Impact of sex and age

**Figure 2:**
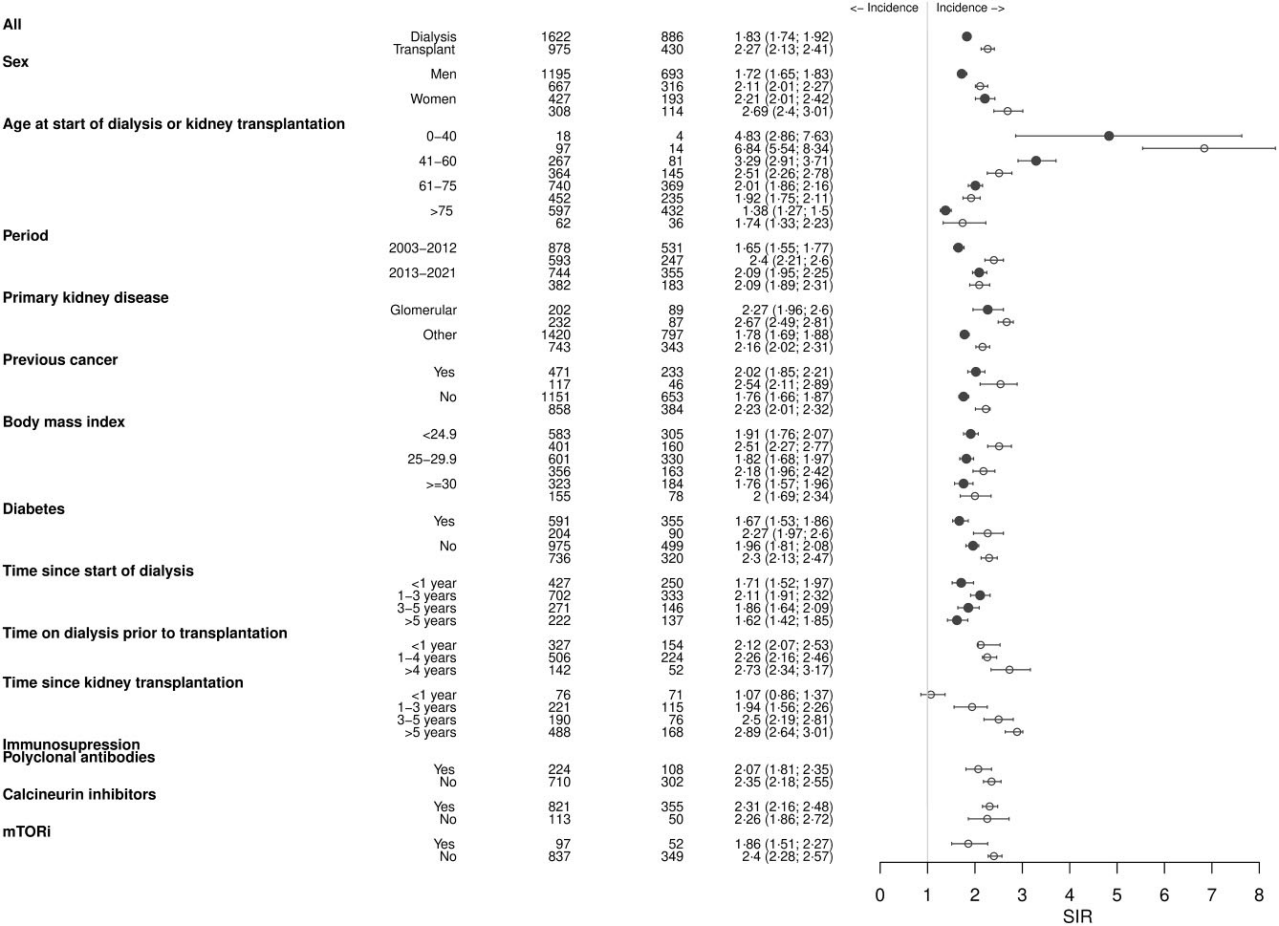
Risk of all-site cancer (excluding non-melanoma skin cancer) in people on dialysis and kidney transplant recipients compared with the general population, by demographics, comorbidities and received treatments.

Although the risk of cancer was significantly higher in both sexes, women experienced a higher excess cancer risk during dialysis and after kidney transplantation compared with men (Fig. 2).

There was an inverse relationship between cancer excess risk and age in both people on dialysis and kidney transplant recipients: the youngest individuals had the highest excess risk, which decreased with age. Consequently, older people with kidney failure had a cancer risk almost comparable to that of the general population (Fig. 2).

### Impact of comorbidities and received treatment

During dialysis, the SIR for all-site cancer excluding non-melanoma skin cancer was higher in individuals with glomerular diseases compared with those with other causes of kidney failure, in people with previous cancers compared with those without, and in individuals from the second half of the cohort (period between 2013 and 2021). In contrast, the risk remained stable for those with diabetes or obesity. There was no trend in SIR with increasing duration of dialysis (Fig. 2).

In the kidney transplant cohort, the SIR also increased for individuals with glomerular diseases but remained stable for those with diabetes, obesity, or previous cancer. There was a tendency for an increased cancer risk with longer dialysis vintage, and with increasing time since kidney transplantation. The SIR did not vary between the first and second halves of the cohort, nor in recipients receiving polyclonal antibodies or calcineurin inhibitors. Interestingly, those treated with mammalian target of rapamycin inhibitors (mTORis) had a reduced excess risk compared with those who did not receive mTORis during the first 6 weeks after kidney transplantation (Fig. 2).

## DISCUSSION

We described the epidemiology of cancer in 21 595 people on dialysis and 8037 kidney transplant recipients over a 19-year period in Catalonia. Our findings revealed a >2-fold increase in cancer risk in people on dialysis and a nearly 4-fold higher risk in kidney transplant recipients compared with the general Catalan population. These risks varied by cancer site, with the highest cancer risks observed in the youngest, those with glomerular diseases and those with the longest time since transplantation.

There are several factors that can contribute to the increased cancer incidence. Kidney disease and cancer share several risk factors, including smoking, metabolic syndrome, and environmental exposures. Additionally, kidney failure is associated with systemic inflammation and acquired immunodeficiency [19], and some people even receive additional immunosuppressive agents to treat the cause of their kidney disease or after transplantation [2]. The higher cancer risk in kidney transplant recipients, despite having fewer comorbidities than those on dialysis and undergoing a thorough screening process while on the waiting list, argues in favor of an important role of immunosuppression after kidney transplantation.

Some cancers with the greatest excess risks are linked to infections, such as human papillomavirus in oropharyngeal and gynecological cancers, and Epstein–Barr virus in lymphoma, reflecting impaired immune control of oncogenic viruses. The increased risk of non-melanoma skin cancer is also well documented, especially in the transplant population. Some types of non-melanoma skin cancer have also been linked to human papillomavirus, although the main risk factor for this cancer is exposure to ultraviolet radiation, and chronic immunosuppression potentiates its carcinogenic effect [20]. However, increased risks are also observed for cancers without known infectious agents.

Kidney cancer might be related to the cyst transformation in end-stage kidneys [21], and partially to enhanced surveillance through routine ultrasounds [22]. The excess risk of thyroid cancer is not well understood but may also involve surveillance bias from imaging patients with hyperparathyroidism [22]. Melanoma and multiple myeloma are also more common in people with HIV infection, suggesting a role for impaired immune surveillance or chronic inflammation [23]. In contrast, breast or prostate cancers, which are common in the general population, occurred at a similar or even lower rate in the kidney failure population. These results are consistent with findings from cohorts in other regions [4, 5, 7] and it has been hypothesized that the patterns may reflect the effect of screening programs in the general population.

Overall, the excess risk pattern we observed in our cohort aligns with findings from other regions. The most notable difference was for melanoma, with more than double the excess risk in both people on dialysis and kidney transplant recipients compared with cohorts from Australia, northern Europe, and USA [4, 5, 7, 24]. The other differences were more modest. However, comparing findings between regions can be challenging due to variations in study periods or cancer registry methodologies. Differences in the management of individuals with kidney failure, environmental risk factors, and cancer incidence in the respective general population could also partially explain variations in excess cancer risk.

Excess risks for all-site cancer (excluding non-melanoma skin cancer) varied among the kidney failure cohort. Identifying individuals with higher excess risk could help provide tailored screening strategies. Younger individuals had the highest excess risks compared with the general population, where the expected number of cancer cases is low. Excess risks decreased with age in the kidney failure cohort, reflecting the increase in risk associated with aging in the general population. Those with glomerular diseases experienced higher excess risks, possibly linked to chronic inflammation or prolonged immunosuppressive therapy [25]. Interestingly, previous cancers did not impact the excess risk in kidney transplant recipients. Contemporary population-based studies report lower cancer recurrence rates compared with earlier reports [26]. Conservative waiting times before kidney transplantation or less aggressive immunosuppressive therapy may contribute to low recurrence rates [27]. However, we could not differentiate between recurrences and new primary cancers or assess the impact of staging or long-term immunosuppression effects.

In kidney transplant recipients, excess cancer risk increased with time since transplantation. During the first year, we did not observe an excess risk compared with the general population, likely due to thorough cancer screening and careful patient selection. From the first year onward, excess risks increased progressively, which may reflect the cumulative effects of immunosuppression. There was also a tendency towards an increased risk of cancer with longer dialysis vintage in the transplant cohort, although not statistically significant. Conversely, excess risks remained stable over time in the dialysis cohort. These patterns are concordant with previous cohorts [4].

There is supporting evidence that mTORis can suppress neoplastic progression in certain malignancies [28], though their effect on overall cancer risk is unclear [29, 30]. In our cohort, we observed a lower excess risk of cancer in those receiving mTORis (excluding non-melanoma skin cancer) during the first 6 weeks. However, as our primary objective was not to evaluate the impact of immunosuppression, and we only had data on treatments received during the first 6 weeks after transplantation, results should be interpreted with caution.

People who started dialysis between 2013 and 2021 exhibited a higher risk of cancer than those who started dialysis during the previous period (2003–12). One potential explanation is the changing baseline characteristics of dialysis patients over time, with an increasing prevalence of comorbidities such as diabetes or previous cancers (Supplementary Table 2). It is also important to note that there were differences in registry coverage and data collection methods between these periods, which may have influenced the observed increase in risk. However, no such differences in cancer risk were observed between the two periods in the kidney transplant cohort, suggesting that variations in cancer data collection have not played a major role.

This study has some limitations. We could not assess some risk factors, such as smoking, socioeconomic factors, changes in immunosuppression after transplantation, or treatments before kidney failure. Also, we had to group specific cancer sites when estimating SIRs due to the nature of the data available, and we lacked information on cancer staging. Third, there were varying timeframes and population coverage between cancer registries, and we cannot rule out that this may have influenced temporal trends. Fourth, we could not differentiate between recurrences and new primary cancers, which may inflate cancer incidence in cancer survivors. Finally, the kidney failure population is at risk of surveillance bias, which could lead to an overestimation of cancer incidence.

However, this study also has several strengths. It represents the largest cohort to examine cancer epidemiology in people on dialysis and kidney transplant recipients in southern Europe, with contemporary data spanning 19 years of follow-up. The Catalan Kidney Registry upholds a high level of quality, offering granular data on comorbidities, along with nearly complete population coverage. Data linkage with the additional cancer databases ensured comprehensive information on cancer diagnoses in this population.

In conclusion, people with kidney failure face a high burden of cancer, particularly after kidney transplantation. Understanding the epidemiology of cancer in the kidney failure population of our region is crucial for identifying individuals at higher risk of cancer and shaping tailored health policies.

## Supplementary Material

sfaf077_Supplemental_File

## Data Availability

Patient-level data from this analysis is protected by national privacy laws. Summary data can be made available upon reasonable request, subject to ethical oversight.
