# Supplementary material for: Cancer risks in people on dialysis and kidney transplant recipients: a Catalan cohort study, 2003–21
Source: Clin Kidney J. 2025 Mar 20;18(4):sfaf077. doi: 10.1093/ckj/sfaf077 (PMC11986820; doi:10.1093/ckj/sfaf077)
Supplement: sfaf077_Supplemental_File [file sfaf077_supplemental_file.docx]

**Supplementary tables and table legends**

Supplementary table 1. Risk of all-site and site-specific cancer in people on hemodialysis and peritoneal dialysis compared to the general population.

| **Site** | **Hemodialysis** | | | | | **Peritoneal dialysis** | | | | |
| --- | --- | --- | --- | --- | --- | --- | --- | --- | --- | --- |
|  | Obs | Exp | SIR | LCI | UCI | Obs | Exp | SIR | LCI | UCI |
| Lip, mouth and pharynx | 60 | 21 | 2.86 | 2.19 | 3.69 | 6 | 2 | 2.67 | 0.98 | 5.82 |
| Esophagus | 16 | 8 | 1.92 | 1.10 | 3.12 | 1 | 1 | 1.12 | 0.03 | 6.25 |
| Stomach | 35 | 30 | 1.17 | 0.82 | 1.63 | 4 | 3 | 1.45 | 0.40 | 3.72 |
| Colon, rectum, and anus | 166 | 149 | 1.12 | 0.95 | 1.30 | 18 | 14 | 1.25 | 0.74 | 1.97 |
| Liver | 30 | 24 | 1.24 | 0.83 | 1.76 | 5 | 2 | 2.10 | 0.68 | 4.89 |
| Gallbladder and biliary tract | 15 | 11 | 1.39 | 0.78 | 2.29 | 2 | 1 | 2.13 | 0.26 | 7.68 |
| Pancreas | 15 | 30 | 0.51 | 0.28 | 0.84 | 1 | 3 | 0.37 | 0.01 | 2.03 |
| Larynx | 12 | 10 | 1.23 | 0.63 | 2.15 | 4 | 1 | 3.47 | 0.95 | 8.89 |
| Trachea, bronchus, and lung | 183 | 96 | 1.91 | 1.64 | 2.21 | 16 | 10 | 1.60 | 0.91 | 2.59 |
| Skin, melanoma | 41 | 15 | 2.74 | 1.97 | 3.72 | 4 | 2 | 2.57 | 0.70 | 6.58 |
| Skin, non melanoma | 728 | 300 | 2.42 | 2.25 | 2.61 | 98 | 27 | 3.57 | 2.90 | 4.35 |
| Breast | 63 | 47 | 1.33 | 1.02 | 1.70 | 6 | 5 | 1.24 | 0.46 | 2.71 |
| Gynecological | 54 | 20 | 2.73 | 2.05 | 3.57 | 8 | 2 | 4.13 | 1.78 | 8.14 |
| Prostate | 140 | 174 | 0.80 | 0.68 | 0.95 | 20 | 19 | 1.06 | 0.65 | 1.63 |
| Testis | 1 | 1 | 1.19 | 0.04 | 6.63 | 1 | 0 | 8.03 | 0.24 | 44.72 |
| Kidney | 192 | 33 | 5.74 | 4.96 | 6.62 | 31 | 4 | 8.84 | 6.00 | 12.54 |
| Bladder and urinary tract | 148 | 53 | 2.79 | 2.36 | 3.27 | 23 | 5 | 4.58 | 2.90 | 6.86 |
| Brain and central nervous system | 15 | 12 | 1.21 | 0.68 | 2.00 | 0 | 1 | 0.00 | 0.00 | 2.33 |
| Thyroid | 16 | 5 | 3.20 | 1.83 | 5.20 | 1 | 1 | 1.53 | 0.05 | 8.51 |
| Lymphoma | 64 | 28 | 2.30 | 1.78 | 2.94 | 9 | 3 | 3.15 | 1.44 | 5.97 |
| Leukemia | 39 | 24 | 1.64 | 1.16 | 2.24 | 6 | 2 | 2.70 | 0.99 | 5.88 |
| Myeloma | 50 | 18 | 2.73 | 2.03 | 3.60 | 3 | 2 | 1.68 | 0.35 | 4.92 |
| Other (excluding skin) | 89 | 55 | 1.63 | 1.31 | 2.00 | 6 | 5 | 1.24 | 0.45 | 2.69 |
| All cancers | 2175 | 1058 | 2.06 | 1.97 | 2.14 | 273 | 103 | 2.65 | 2.35 | 2.99 |

*Exp, expected cases; LCI, lower confidence interval; Obs, observed cases; SIR, standardized incidence ratio; UCI, upper confidence interval*.

Supplementary table 2. Demographics of people on dialysis by year of dialysis initiation.

|  | **2003-2012** | **2013-2021** |
| --- | --- | --- |
| Total | 10,342 | 11,253 |
| Median age (years, IQR) | 68 [55, 77] | 70 [58,78] |
| Diabetes (n, %) | 3,441 (33.3) | 4,602 (40.9) |
| Previous cancer (n, %) | 1,836 (17.8) | 3,244 (28.9) |
| Primary kidney disease |  |  |
| Glomerular | 1,503 (14.5) | 1,398 (12.4) |
| Diabetes | 2,293 (22.2) | 2,594 (23.1) |
| Hypertension/renal artery disease | 1,734 (16.8) | 1,291 (11.5) |
| Tubulointerstitial/urologic | 887 (8.6) | 777 (6.9) |
| Polycystic kidney disease | 716 (6.9) | 633 (5.6) |
| Unknown cause/other | 3,167 (30.6) | 3,800 (33.8) |
| *Missing* | 42 (0.40) | 760 (6.7) |

*IQR, interquartile range*
